# Intra-articular injection of clioquinol ameliorates osteoarthritis in a rabbit model

**DOI:** 10.3389/fmed.2022.1028575

**Published:** 2022-11-17

**Authors:** Xiaoqing Wu, Peng Xu, Xuanren Shi, Jian Shang, Xiaoyong Chen, Anyun Guo, Fuming Wang, Zhanhai Yin

**Affiliations:** ^1^Department of Orthopaedics, The First Affiliated Hospital of Xi’an Jiaotong University, Xi’an, China; ^2^Department of Orthopaedics and Traumatology, Shenzhen University General Hospital, Shenzhen, China; ^3^Department of Joint Surgery, Xi’an Honghui Hospital, Xi’an Jiaotong University Health Science Center, Xi’an, China; ^4^Department of Hematology and Oncology, Shenzhen University General Hospital, Shenzhen, China

**Keywords:** osteoarthritis, clioquinol, intra-articular injection, autophagy, therapeutic potential

## Abstract

Osteoarthritis (OA) is characterized by the degeneration of articular cartilage. Decreased autophagy is tightly associated with chondrocyte death, which contributes to the progression of OA. Thus, pharmacological activation of autophagy may be a promising therapeutic approach for OA. Here, we discovered that clioquinol, an antibiotic, significantly induces autophagy in OA chondrocytes from human tissue and rabbit model. Meanwhile, clioquinol can also augment the expression of extracellular matrix (ECM) components and suppress inflammatory mediators to improve OA microenvironment. Intra-articular injection of clioquinol can greatly prevent or slow down the development of this disease in a trauma-induced rabbit model of osteoarthritis. Such protective effect induced by clioquinol was at least in part explained by decreasing chondrocyte apoptosis and increasing autophagy. This study reveals the therapeutic potential of clioquinol in OA treatment.

## Introduction

Osteoarthritis (OA) is the most common degenerative disease worldwide characterized by the loss of chondrocytes, degradation of articular matrix, and synovial inflammation (1). The etiology of OA is multifactorial and complex, with a variety of risk factors contributing to the progression, such as mechanical stress, aging, obesity, inflammation, and genetic susceptibility (2). Articular cartilage is composed of the extracellular matrix (ECM), mainly containing collagen type II and the cell type—chondrocytes. Cartilage architecture and biochemical composition are regulated by chondrocytes in response to the alterations from the surroundings of cartilage matrix (1). The ability of adult articular chondrocytes to maintain the normal cartilage matrix architecture is limited and declines with age (3). Therefore, keeping the chondrocytes in a healthy state may be an important way for maintaining the integrity of the entire cartilage.

Autophagy is a highly conserved physiological process that degrades long-lived or impaired organelles and proteins via the lysosomal system (4). Increasing evidence has shown that autophagy plays an important role in cell survival, aging, and homeostasis (5). In addition, it has been revealed that autophagy is associated with the pathogenesis of OA (6). During the development of OA, autophagy may function as an adaptive response to exert protective effect against various environmental changes, while the failure of the adaptation may lead to the progression in cartilage degradation (7). Previous studies found that the intra-articular injection of rapamycin, an autophagy inducer, facilitates the postponement of cartilage degeneration in the OA mouse model (8). Thus, it can be concluded that pharmacological activation of autophagy may be a promising therapeutic strategy for OA.

Herein, we attempt to explore new autophagy-inducing pharmacophores. Previous research revealed that some antibiotics can be utilized to induce autophagy (9, 10). Clioquinol (5-chloro-7-iodo-8-quinolinol), an antimicrobial agent against common pathogenic microbes, functions as a novel autophagy activator in multiple cells (11–13). Clioquinol can trigger pro-death autophagy via interrupting mTOR signaling pathway in leukemia and myeloma cells, which not only inhibits enzymatic activity of mTOR (a critical modulator of autophagy) as rapamycin, but also suppresses the expression of mTOR (12). Clioquinol also induces autophagy in a zinc-dependent manner and contributes to the clearance of aggregated proteins in astrocytes and neurons (11). The protective effects of clioquinol have been reported in various neurodegenerative diseases, such as Alzheimer’s disease (14), Parkinson’s disease (15), and Huntington’s disease (16), but not yet in OA. Therefore, exploring the potential of clioquinol as autophagy inducer in OA treatment may be helpful for the therapy.

In this study, we investigated the effects of clioquinol on autophagy process in chondrocytes. Furthermore, we also evaluated the therapeutic potential of clioquinol in the rabbit OA model of anterior cruciate ligament transection with partial medial meniscectomy (ACLT + PMM) and explored the underlying mechanism.

## Materials and methods

### Isolation and culture of chondrocytes

Human cartilage tissue was obtained from the knees of OA patients who had undergone Total Knee Arthroplasty (TKA). This study was approved by the human research ethics committee. All patients’ OA satisfy the American College of Rheumatology’s criteria for OA (17), and the patients’ consent was obtained. The Clinical characteristics of OA patients are in Table 1. The isolation and culture of chondrocytes were applied according to the previous study (10). A non-weightbearing area of cartilage without any macroscopically visible abnormalities was harvested and washed in sterilized saline. Next, the tissue was cut into smaller sections of 1 mm^3^ and digested by trypsin (2.5 mg/ml) (Sigma Co., St. Louis, MO, USA) at 37°C for 40 min, and then treated for 8 h with Type II collagenase (2 mg/ml) (Sigma Co., St. Louis, MO, USA) in a DMEM/F12 medium (Thermo Scientific, Waltham, MA, USA). The isolated chondrocytes were placed in DMEM/F12 with 10% FBS and 100 units/ml of penicillin and 0.1 mg/ml of streptomycin, incubated at 37°C. Following the above procedure, we applied the second-passage chondrocytes for further studies.

**TABLE 1:** Clinical characteristics of osteoarthritis (OA) patients.

Sample	Sex	Age (year)	Disease duration (year)	CRP (μg/ml)	ESR (IU/ml)	RF (IU/ml)	Sample-obtained time
OA1	Male	65	16	7.31	18	10.44	February 2021
OA2	Male	72	13	5.23	24	16.32	February 2021
OA3	Male	71	17	2.45	19	4.75	March 2021
OA4	Male	63	18	5.78	7	9.87	March 2021
OA5	Male	69	13	2.12	16	15.27	March 2021
OA6	Female	64	20	7.98	27	17.21	March 2021
OA7	Female	55	9	9.12	25	13.56	April 2021
OA8	Female	63	15	11.13	28	12.98	April 2021
OA9	Male	64	7	7.63	15	9.45	May 2021
OA10	Male	72	10	2.78	14	5.32	May 2021

CRP, C-reactive protein; ESR, erythrocyte sedimentation; RF, rtheumatoid factor.

### Cell viability assay

The cell viability of chondrocytes was detected by the CCK-8 Reagent (Thermo Scientific, Waltham, MA, USA) according to the manufacturer’s protocol. Chondrocytes were treated with clioquinol for 24 and 48 h, respectively. The cells were washed with PBS, and then added CCK-8 solution. The 450 nm absorbance was detected by micro-plate reader. All experiments were repeated at least five times.

### Establishment of osteoarthritis model and intra-articular injection of clioquinol

Male white New Zealand rabbits (2.5–2.8 kg, 3-month-old) were used with the approval from the institutional animal care and use committee. Anterior cruciate ligament transection with partial medial meniscectomy (ACLT + PMM) were performed on rabbits as described previously (18). Briefly, under general anesthesia, the anterior horn of the medial meniscus was dissected and the anterior cruciate ligament of the right knee was transected. In the control group, a sham operation was carried out on the contralateral knee with no meniscus dissection and no ligament transection and then treated by the vehicle only. Eighteen rabbits were randomly assigned to three groups (*n* = 6/group): the sham group (sham operation plus the vehicle treatment as control), ACLT + PMM plus the vehicle treatment group, ACLT + PMM plus the clioquinol (5 mg kg^–1^) treatment group (10). Rabbits were given clioquinol (100 μl) were intra-articular injected into rabbits once a week following surgery for 8 weeks. All rabbits were kept in individual cages at 22 ± 3°C with 55 ± 20% humidity and a 12-h light-dark cycle. After 8 weeks of surgery, the tibial plateaus of the rabbits’ hind legs were harvested. Animal studies were based on ARRIVE guidelines.

### Autophagosome formation detection

Chondrocyte autophagosome formation was detected by Cyto-IDTM autophagy detection kit (Enzo Life Sciences, Farmingdale, NY, USA), following the manufacturer’s recommendations. Articular cartilage cells of humans with OA were treated with or without 5 μM clioquinol for 48 h and then stained with a dual detection reagent for 30 min in the dark at 37°C. The green dot fluorescence (autophagosome) was observed under microscope.

### RNA extraction and RT-qPCR analysis

Total RNA was isolated using the TRIzol reagent (Invitrogen, Carlsbad, CA, USA). Real-time RT-qPCR was performed with the Applied Biosystems StepOnePlus Real-Time PCR System. Information on the primer sequences was provided in Table 2. All samples were performed in triplicate.

**TABLE 2:** Details of the primers used in RT-qPCR.

Gene name	Sense (5′–3′)	Antisense (5′–3′)
*Col2a1*	GGCAATAGCAGGTTCACGTACA	CGATAACAGTCTTGCCCCACTT
*Acan*	TCGAGGACAGCGAGGCC	TCGAGGGTGTAGCGTGTAGAGA
*MMP-1*	GGGGCTTTGATGTACCCTAGC	TGTCACACGCTTTTGGGGTTT
*MMP-13*	ACTGAGAGGCTCCGAGAAATG	GAACCCCGCATCTTGGCTT
*IL-6*	CCACTCACCTCTTCAGAACGAAT	GGCAAGTCTCCTCATTGAATCCA
*IL-1ß*	AACAGGCTGCTCTGGGATTC	GGTCGGAGATTCGTAGCTGG
*LC3*	CCACACCCAAAGTCCTCACT	CACTGCTGCTTTCCGTAACA
*Beclin1*	AAATGCTGCTTGGGGTCAGA	CGGAATCCACCAGACCCATA
*ATG5*	AAGCAACTCTGGATGGGATT	GCAGCCACAGGACGA AAC
*ATG7*	CAGTCCGTTGAA GTCCTC	TCAGTGTCCTAGCCACATTAC
*GAPDH*	AGGTCGGTG TGAACGGATTTG	GGGGTCGTTGATGGC AACA
*DRAM1*	TCAAATATCACCATTGATTTCTGT	GCCACATACGGATGGTCATCTCTG

### Western blot

The treated cells were collected and lysed for protein sample preparation. We loaded 30 mg of lane protein into the well of SDS page gel and electrophoretically transferred the protein to NC membranes (Millipore, Billerica, MA, USA). The membranes were blocked for 1 h and incubated in primary antibodies against microtubule associated protein 1 LC3 (1:1,000, Sigma Co., St. Louis, MO, USA), p62 (1:8,000, Abcam, Cambridge, UK), and β-actin (1:1,000, Bioss, Beijing, China). The membranes were then washed and incubated with secondary antibodies (1:10,000, Bioss, Beijing, China) for 2 h. Afterward, the membrane was then washed and exposed with an electrochemiluminescence system. Relative densitometric analysis (a semi-quantitative analysis) was performed following densitometric scanning.

### Immunohistochemistry

After the tissues were dewaxed, 3% H_2_O_2_ was applied to suppress the levels of endogenous peroxide on the histologic slices, after which microwave heating was used to retrieve antigen. The slices were blocked for 0.5 h with goat serum (Beyotime Institute of Biotechnology, Shanghai, China). The slices were then incubated in the solution of MMP-13 primary antibodies (1:100, Abcam, Cambridge, UK), Col-2a1 antibodies (1:100, Abcam, Cambridge, UK), Cleaved-caspase3 antibody (1:200, Abcam, Cambridge, UK). After the incubation, a horseradish peroxidase-conjugated secondary antibody (Abcam) was used for 0.5 h, and cells were stained with 3,3′- diaminobenzidine (Beyotime Institute of Biotechnology) and mounted. The microscope was used for detecting the percentage of MMP-13, Cleaved-caspase3 positive cells (brown cells).

### Transmission electron microscopy

Chondrocytes or cartilage tissue were fixed in ice-cold 2% glutaraldehyde/0.1 M PBS and post-fixed in 1% osmium tetroxide. After being washed and dehydrated with a series of graded ethanol (30–100%), the samples were embedded in propylene oxide or embedding resin (1:1). Resin blocks were cut into thin sections, and the sections were placed on copper grids and stained with uranyl acetate and lead citrate. An H-7650 transmission electron microscope was used for detecting the autophagic vesicles (double membrane-enclosed vesicles containing engulfed organelles or other cell components).

### Histological assessment

The tibial plateaus of hind legs from rabbits were fixed in 4% paraformaldehyde and decalcified for 2 months with 10% EDTA. The tissues were then dehydrated, infiltrated with paraffin, and embedded in paraffin wax. The paraffin blocks were sectioned into 5 μm slices along the sagittal plane using a microtome. Safranin O-fast green and alcian blue staining was performed. Select three slices from each medial tibial plateau, and two observers who were blinded to animal study, respectively, utilized a semi-quantitative scoring system (OARSI’s histopathology grading system of cartilage OA) to assess articular cartilage degeneration.

### Statistical analysis

All the data are presented as mean ± SD. Differences between two groups were analyzed by the unpaired *t*-test or the Mann-Whitney U test. Differences between different groups were analyzed by ANOVA. *P*-values less than 0.05 were statistically significant.

## Results

### Clioquinol induces autophagy in human osteoarthritis chondrocytes

**FIGURE 1:**
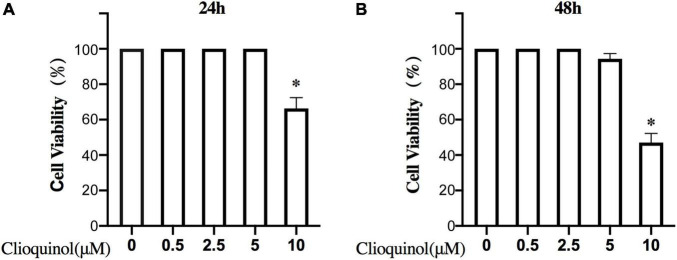
Effects of clioquinol on the cell cytotoxicity *in vitro*. The cytotoxic effects of clioquinol on chondrocytes were determined with increasing concentrations (0, 0.5, 2.5, 5, and 10 μM) for 24 and 48 h using a CCK8 assay **(A,B)**. All experiments were repeated five times. All data are shown as the mean ± SD. *p < 0.05.

**FIGURE 2:**
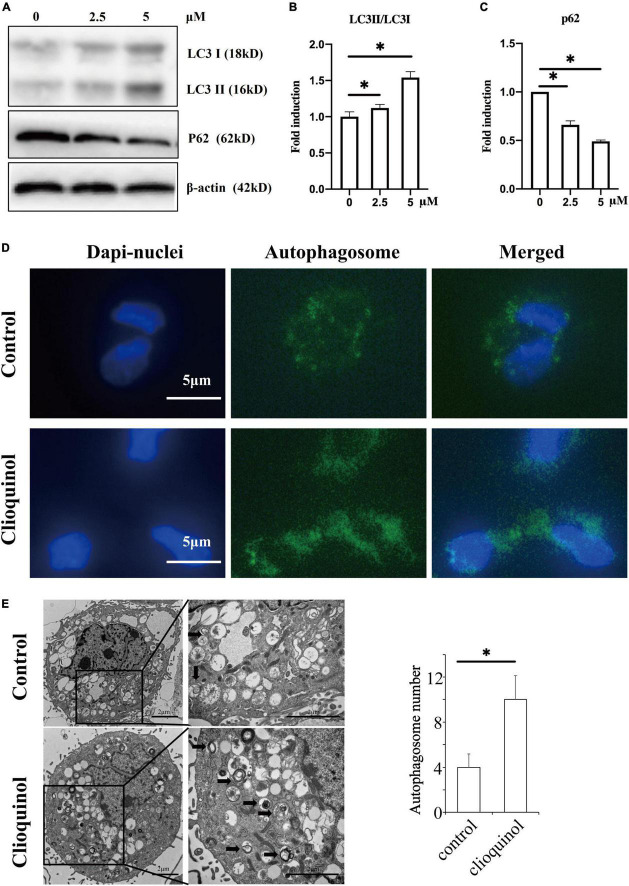
Clioquinol induces autophagy in human osteoarthritis (OA) chondrocytes. The expression of LC3, p62, and β-actin were detected with Western blotting **(A–C)**. Human OA chondrocytes were treated or not treated with 5 μM of clioquinol for 48 h, and autophagosome formation (green dots) was detected using an autophagy detection kit **(D)** and transmission electron microscopy **(E)**. All data are shown as the mean ± SD. *p < 0.05.

First, we investigated the cytotoxic effect of clioquinol on human OA chondrocytes. The cells were incubated with clioquinol at different concentrations (0, 2.5, 5, and 10 μM) for 24 and 48 h. Finally, CCK-8 analysis showed that no apparent cytotoxic effects on chondrocytes were observed at low concentrations of clioquinol (0–5 μM) (Figures 1A,B). However, at 10 μM, clioquinol induced a modest level of cell death. Based on this result, we chose 5 μM as the concentration for following studies. To determine whether clioquinol could induce autophagy, western blot was performed to detect the marker of autophagy, LC3 and P62. The clioquinol-treated chondrocytes show an increased level of LC3-I/II, and a decreased P62 level, in a concentration-dependent manner (Figures 2A–C), indicating the augment of autophagy. Other autophagy-associated genes, DRAM1, ATG5, and ATG7, were also dose-dependently increased (Supplementary Figure 1). Furthermore, we also used immunofluorescence staining (Figure 2D) and transmission electron microscopy (Figure 2E) to visualize the autophagosome formation. In consistent with the results of western blot, autophagosome formation was enhanced in the clioquinol-treated chondrocytes, compared to the control. Together, these results indicated that clioquinol exposure could facilitate autophagy in human OA chondrocytes.

### Clioquinol enhances chondrogenic markers and reduces inflammatory markers in human osteoarthritis chondrocytes

**FIGURE 3:**
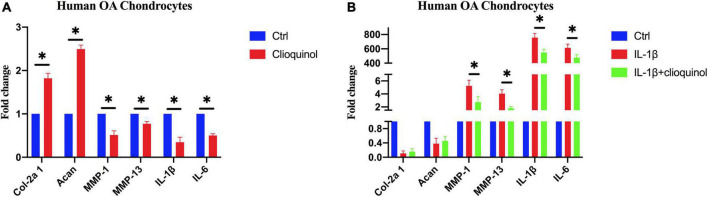
The expression of osteoarthritic markers in human osteoarthritis (OA) chondrocytes after incubating with clioquinol (5 μM) **(A)**, or IL-1ß (10 ng/ml), IL-1ß (10 ng/ml) + clioquinol (5 μM) **(B)**, determined by PCR. All data are shown as the mean ± SD. *p < 0.05.

We next sought to characterize the osteoarthritic microenvironment after clioquinol treatment. RT-qPCR results showed that the mRNA levels of chondrogenic marker Col-2a1 and Acan were significantly elevated after the exposure to clioquinol (Figure 3A). Meanwhile, clioquinol treatment can also reduce the levels of inflammatory marker MMP-1, MMP-13, IL-1ß, and IL-6 (Figure 3A). Additionally, clioquinol can also exert preventive effects on the function of IL-1ß, an important inflammatory factor. Although clioquinol failed to rescue the IL-1ß-induced inhibitory effects on Col-2a1 and Acan, it correspondingly repressed the IL-1ß-mediated upregulated expression of MMP-1, MMP-13, IL-1ß, and IL-6 (Figure 3B).

### Clioquinol ameliorates osteoarthritis development in a rabbit model

**FIGURE 4:**
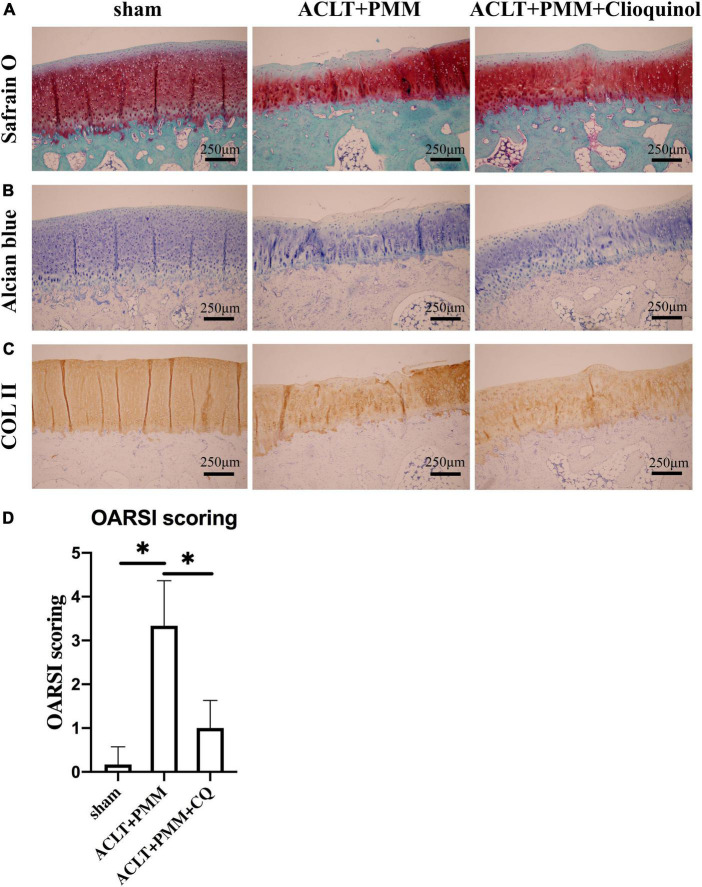
Clioquinol ameliorates osteoarthritis (OA) development in rabbit model. **(A,B)** Representative Safranin O-Fast green and Alcian Blue staining of cartilage in three groups (*n* = 6) at the 8th weeks post-surgery. **(C)** Representative IHC staining showing the expression and distribution of Col-2a1 in cartilage after treatment. **(D)** Cartilage degeneration evaluated by OARSI scoring system. All data are shown as the mean ± SD. *p < 0.05.

We further investigated the therapeutic efficacy of clioquinol on ACLT + PMM-induced OA *in vivo*. Clioquinol was administered by intra-articular injection once a week for 8 weeks beginning the day after surgery. Histological analysis by Safranin O and Alcian Blue staining respectively showed osteoarthritic changes with cartilage abrasion and hypocellularity in the ACLT + PMM group, whereas no OA-like changes were observed in the sham group (Figures 4A,B). However, the ACLT + PMM + clioquinol group showed less cartilage erosion and richer proteoglycan (Figures 4A,B), suggesting clioquinol ameliorates ACLT + PMM-induced impairment. The contrast in different groups of Col-2a1 expression was not obvious, yet the abrasion in the control group was profound (Figure 4C). Consistent with staining, OARSI score of the ACLT + PMM group was significantly higher than that of the sham group, while the clioquinol treatment resulted in the decrease of OARSI scores (Figure 4D).

### Clioquinol reduces the expression of inflammatory markers in the rabbit model

**FIGURE 5:**
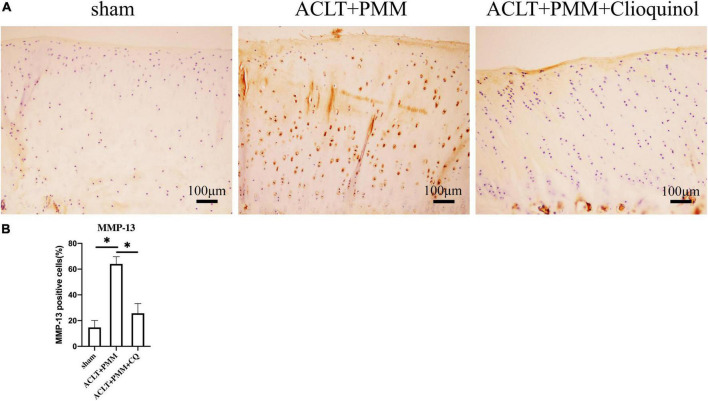
The change of MMP-13 expression in cartilage after intra-articular injection of clioquinol. **(A)** Representative IHC staining showing the expression and distribution of MMP-13 in cartilage. **(B)** Quantitation of ICH staining. All data are shown as the mean ± SD. *p < 0.05.

Immunohistochemistry (IHC) as performed for MMP-13, a main protease responsible for collagen degradation in articular cartilage. The results showed that chondrocytes with high MMP-13 expression were increased in the ACLT + PMM group compared to the sham group. However, in the ACLT + PMM + clioquinol group, MMP-13 positive cells in the articular cartilage was significantly reduced (Figures 5A,B).

### Clioquinol enhances autophagosome formation and attenuates apoptosis of chondrocytes in the rabbit model

**FIGURE 6:**
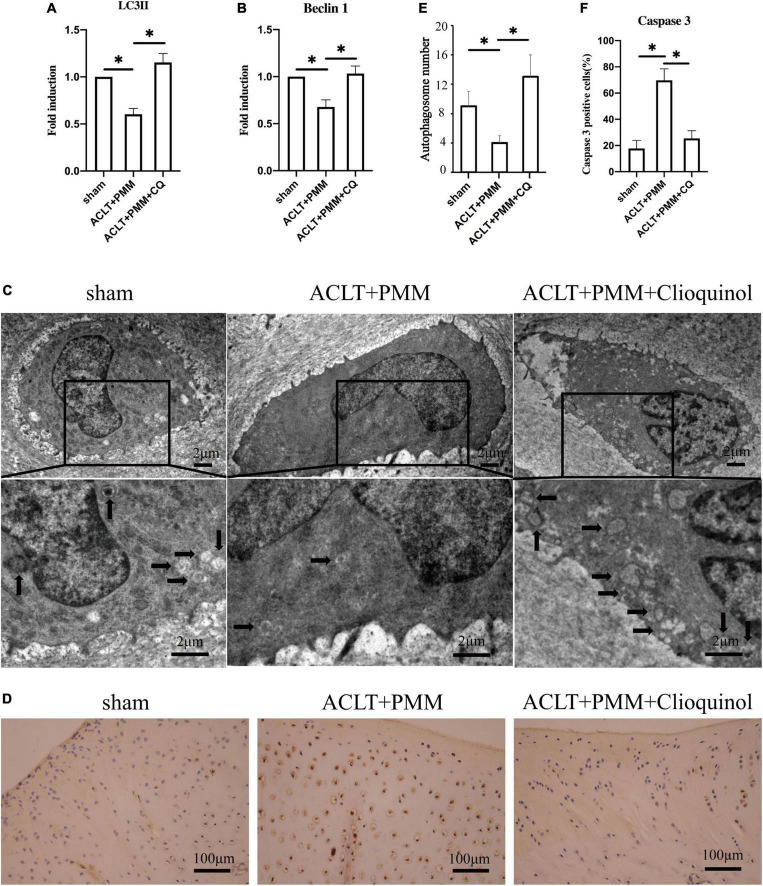
**(A,B)** The expression of LC3 and Beclin-1 evaluated by PCR. **(C,E)** Autophagosome formation (arrows) detected using transmission electron microscopy. **(D)** Representative IHC staining showing the expression and distribution of cleaved caspase 3 in cartilage. **(E,F)** Quantitative analysis of autophagosome formation and cleaved caspase 3. All data are shown as the mean ± SD. *p < 0.05.

We examined the expression of autophagy markers to clarify if clioquinol treatment can facilitate autophagy in the rabbit OA model. RT-qPCR results showed that the mRNA expression of LC3 and beclin-1 was significantly (*p* < 0.05) reduced in rabbit OA chondrocytes compared with the sham surgery chondrocytes, while clioquinol treatment markedly rescued the inhibited expression of these autophagy markers (Figures 6A,B). Consistent with the expression of autophagy markers, TEM also showed that autophagosome formation was significantly augmented in the ACLT + PMM + clioquinol group, compared with the ACLT + PMM group (Figures 6C,E). Apoptosis in chondrocytes is positively associated with OA progression. Herein, we also detected the level of apoptosis marker cleaved-caspase 3 by immunohistochemical staining. The cartilage of ACLT + PMM group showed an increased expression of cleaved caspase 3 compared to the sham group, while clioquinol administration significantly repressed the ACLT + PMM surgery-induced elevation of cleaved caspase 3 (Figures 6D,F), which indicates clioquinol can also protect chondrocytes from the apoptosis in OA progression.

## Discussion

Osteoarthritis has been regarded as a degenerative disease with a high prevalence, which is the primary cause of disability and burden for the elderly (19, 20). There is a clear and urgent need to develop new drugs for OA treatment. In this study, we reported that clioquinol can significantly promote autophagy, enhance chondrogenic markers and inhibit inflammatory markers in OA chondrocytes. Furthermore, we demonstrated that the intra-articular clioquinol can ameliorate OA damages in the ACLT-induced OA rabbit model, and clioquinol can also block chondrocyte apoptosis, which may be achieved by enhancing autophagy.

Autophagy plays an essential role in maintaining cellular metabolism and homeostasis (21). Recent studies suggest that the imbalance of autophagy is a key factor in the pathogenesis of OA (22). Studies have demonstrated that the level of autophagy in OA cartilage is reduced (23) and autophagy can protect chondrocytes from the degradation (24). Activation of autophagy in chondrocytes by intra-articular injection of resveratrol, an autophagy inducer, can significantly delay articular cartilage degeneration in a destabilized medial meniscus OA mouse model (25). Our previous study found that an intra-articular injection of chloramphenicol attenuates the severity of cartilage degradation in a type II collagen-induced rabbit model of OA, which may be associated with the induction of autophagy (10). Therefore, pharmacological induction of autophagy may be an appropriate therapeutic approach for OA. The development of safe and effective drugs that can enhance autophagic activities or restore autophagy flux is a promising strategy for the treatment of OA. This study, therefore, was planned to assess the potential of clioquinol as autophagy inducer in primary chondrocytes and rabbits with ACLT + PMM surgery-induced OA. Clioquinol is a quinoline derivative used as an antibiotic for the treatment of diarrhea and soft tissue infections (26, 27). Recently, clioquinol has been proven to induce autophagy of a variety of cells, including astrocytes, neurons, leukemia and myeloma cells (11–13). However, there is no previous study to explore the application of clioquinol against OA development, whereas our study revealed the potential of clioquinol as an autophagy inducer for the treatment of OA. From a mechanistic standpoint, previous studies indicated that clioquinol acts as a zinc ionophore and increases intracellular free zinc levels in the cytosol and in lysosomes, which augments autophagic flux (11, 28). However, whether clioquinol would induce autophagy in OA through a similar or the same pathway requires further exploration. In order to investigate if the autophagy induction property of clioquinol can protect chondrocytes, we incubated primary chondrocyte cells with or without clioquinol. Consistent with the previous studies, autophagy is a self-protective process in OA in response to the stimulation by clioquinol. In our study, clioquinol exerts chondroprotective effects on human OA chondrocytes, which was manifested by the increased chondrogenic markers Col-2a1 and Acan and the suppressed expression of genes encoding inflammatory cytokines IL-1β and IL-6, as well as the cartilage-degrading enzymes from the MMP family, including MMP-1 and MMP-13 (Figure 3).

According to *in vitro* experiments, we also sought to investigate whether intra-articular injection of clioquinol could block OA progression *in vivo*, and ACLT + PMM surgery-induced OA rabbit model was constructed for further studies. Our study shows that intra-articular injection of clioquinol can distinctly repress articular cartilage erosion and rescue the proteoglycan content. Increasing evidences show that MMP-13, a zinc-dependent protein, plays a vital role by degrading type II collagen in articular cartilage in OA (29), which indicates that the level of MMP-13 is positively associated with OA severity (30–32). Our findings shows that intra-articular injection of clioquinol can inhibit MMP-13 expression, which was consistent to our *in vitro* study. However, there was no significant degradation of Col-2a1 in the ACLT + PMM group, which may be owing to the long half-life of Col-2a1 and activated metabolism of chondrocytes after the surgery that confound the effect of clioquinol administration on the expression of Col-2a1 (33, 34).

We also investigated the role of an intra-articular injection of clioquinol on autophagy in rabbits with OA and detected that clioquinol can increase the expression of autophagy-related factors, including LC3 and Beclin1. More autophagosomes were observed by transmission electron microscopy, consistent with the alterations of autophagy markers. In addition, clioquinol reduced the level of cleaved caspase 3, a primary executioner for apoptosis, which indicated that the increase of autophagy with a subsequent decrease of apoptosis may be a part of the mechanism of clioquinol-mediated amelioration for OA.

There were also some limitations to our study. First, this is a preliminary result, and it is expected that details of the interaction between the clioquinol-induced autophagy and OA require further research. Second, the animal model we used in this paper was ACLT + PMM surgery-induced OA model, which is a classical method for establishing OA models; thus, it may not be broadly representative of all OA conditions. Third, although clioquinol is promising for the treatment for OA, owing to the side effect–termed subacute myelo-optic neuropathy (SMON), it still need more efforts to explore its appropriate utilization in human.

Taken together, our results suggest that intra-articular injection of clioquinol can alleviate ACLT + PMM surgery-induced OA progression. The underlying mechanisms may include reducing MMP-13, increasing autophagy and decreasing chondrocyte apoptosis. Intra-articular administration of clioquinol may be a promising treatment for OA, and additional comprehensive studies examining the clinical potential of clioquinol for OA therapy are still required.

## Data availability statement

The original contributions presented in this study are included in the article/Supplementary material, further inquiries can be directed to the corresponding author.

## Ethics statement

The studies involving human participants were reviewed and approved by the Human Research Ethics Committee. The patients/participants provided their written informed consent to participate in this study. This animal study was reviewed and approved by the Institutional Animal Care and Use Committee.

## Author contributions

XW, XS, and ZY: conception and design. XW, JS, and XC: analysis and interpretation of the data. XW, XS, AG, PX, and ZY: drafting of the manuscript. PX, JS, FW, and ZY: critical revision of the manuscript for important intellectual content. XW, PX, JS, and ZY: final approval of the manuscript. PX: provision of the study material or patients. XW, XS, AG, and ZY: statistical expertise. AG, PX, and ZY: obtaining of funding. XW, XS, XC, FW, and ZY: administrative, technical, and logistic support. XW, XC, and FW: collection and assembly of data. All authors drafting the manuscript or revising it critically for intellectual content and approved the final version to be published.
