# Supplementary figures and images for: Intra-articular injection of clioquinol ameliorates osteoarthritis in a rabbit model
Source: Front Med (Lausanne). 2022 Nov 17;9:1028575. doi: 10.3389/fmed.2022.1028575 (PMC9712181; doi:10.3389/fmed.2022.1028575)

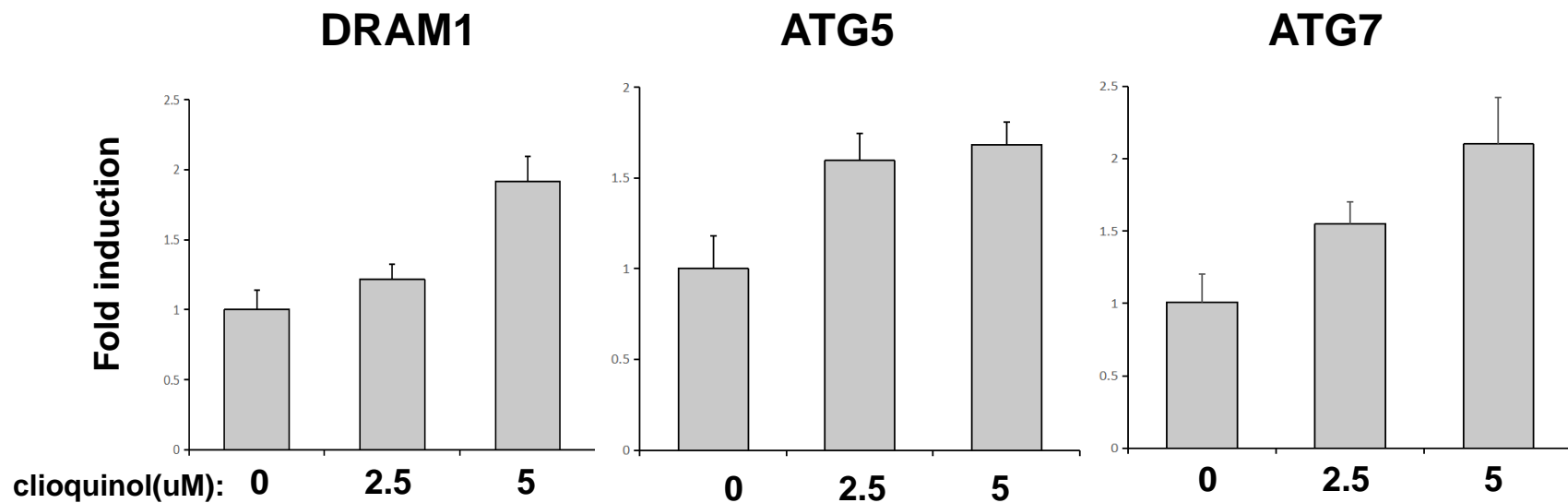

Supplementary Figure 1: The change of autophagy-related genes detected by qPCR.

Supplement: Supplementary file 1 [file Image_1.pdf]
